# Cancer Visibility among Iranian Familial Networks: To What Extent Can We Rely on Family History Reports?

**DOI:** 10.1371/journal.pone.0136038

**Published:** 2015-08-26

**Authors:** Hossein Molavi Vardanjani, Mohammad Reza Baneshi, AliAkbar Haghdoost

**Affiliations:** 1 Research Center for Modeling in Health, Institute for Futures Studies in Health, Kerman University of Medical Sciences, Kerman, Iran; 2 Regional Knowledge Hub, and WHO Collaborating Center for HIV Surveillance, Institute for Futures Studies in Health, Kerman University of Medical Sciences, Kerman, Iran; University of Texas MD Anderson Cancer Center, UNITED STATES

## Abstract

**Objective:**

Patients’ unawareness of their cancer diagnosis (PUAW) and their tendency for non-disclosure (TTND) to relatives leads to a lack of cancer visibility among familial networks. Lack of familial cancer visibility could affect the accuracy of family cancer history (FCH) reports. In this study, we investigated familial cancer visibility and its potential determinants.

**Patients and Methods:**

A sample of patients with a confirmed cancer diagnosis was interviewed. Participants were asked about their number of relatives, number of their relatives who are aware about the cancer diagnosis, and the number of relatives from whom they intended to conceal their diagnosis. PUAW was also assessed. Point estimates and 95% confidence intervals were calculated using the bootstrap technique. Multivariate analyses were conducted using mixed Poisson and logistic regression analyses.

**Results:**

A total of 415 participants with a mean age of 53±15 years and a male to female ratio of 0.53 were enrolled in this study. The rates of PUAW, TTND, and familial cancer visibility in the total sample were 0.20 (95% confidence interval (CI): 0.16, 0.24), 0.16 (95% CI: 0.12, 0.19), and 0.86 (95% CI: 0.83, 0.89), respectively. PUAW (adjusted rate ratio (RR) = 1.32, 95% CI: 1.27, 1.38), TTND (RR = 0.92, 95% CI: 0.91, 0.93), and the patients’ gender (RR = 0.92, 95% CI: 0.82, 0.95) were the most important determinants of familial cancer visibility.

**Conclusion:**

Familial cancer visibility may be a point of concern among the Iranian population. Self-reported cancer histories and FCHs may have low sensitivities (not exceeding 80% and 86%, respectively) in this population. However, these estimates may vary across different societies, because of societal and cultural contexts.

## Introduction

Family cancer history (FCH) reports are a frequently used data source in epidemiological studies [1–3]. They are also crucial in genetic risk assessments and predictive models when making referrals for genetic counseling and inherited cancer risk categorization [4–7]. Although FCHs are widely used and dozens of studies have examined their precision, the accuracy of FCH is still a subject of controversy [8–12]. Recent evidence has shown that, because of misreporting on FCHs, risk prediction models (such as BRCAPRO) could underestimate the risk of a mutation in the BRCA1/BRCA2 genes for hereditary breast and ovarian cancer syndrome [13, 14]. Clinicians, genetic consultants, and preventive specialists have the responsibility to accurately detect inherited cancer risks and encourage familial information transmission [15]. Therefore, in order to improve FCH accuracy, we need to identify its determinants.

Although the evidence has shown a high specificity for FCHs [16], reported values for the sensitivity of FCHs vary widely (from 36 to 92 percent) [8, 16, 17]. Therefore, FCH reports mainly suffer from false negatives (FN) rather than false positives.

A FN FCH may be due to a lack of accuracy in patient self-reports, a lack of awareness about his/her relative’s cancer diagnosis, and a lack of closeness to the affected relative, as well as the patients’ recall bias, age, and gender [9, 16, 18].

The accuracy of self-reporting has been addressed in several studies and self-reports are often compared to medical records or cancer registry data (the gold standards) [19–21]. In addition, a recent study by Inoue et al. [22] reported an overall FN rate of 47% for self-reported cancer history. An FN self-report may be the result of the patients’ unawareness about her/his cancer diagnosis (PUAW) or recall bias [19–21, 23, 24]. PUAW, in turn, is the result of a physician’s reticence to give patients their diagnosis. Therefore, non-disclosure to patients might be a distal determinant of FCH accuracy.

**Fig 1:**
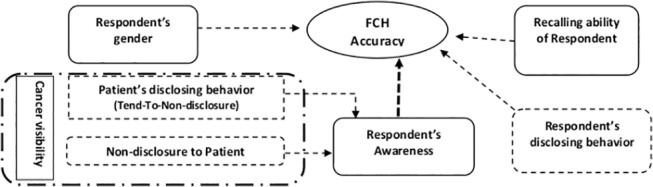
Schematic web of the determinants for the accuracy of family cancer history reports. Solid line box: Previously reported determinants. Dashed arrow: Potential association. Long dash-dotted line box: What we investigated in this study. Dashed line box: Hypothetical determinants

In order to examine the factors associated with lack of FCH accuracy, we mapped a web of known determinants. The hypothesized factor, tendency for non-disclosure (TTND), was incorporated into the web (Fig 1). The tendency for non-disclosure (TTND) of a given patient represents the tendency of a patient to not disclose her/his cancer to relatives, which is the result of cultural issues such as cancer stigmatization or desire to protect loved ones [25–27]. Therefore, we believe that the tendency for non-disclosure (TTND) may be a determinant of lack of FCH accuracy.

Social scientists believe that the TTND results in a visibility bias for population size estimation efforts [28, 29]. This bias causes us to underestimate the size of hard-to-count populations [30]. Inspired by sociologists’ jargon, a FN FCH may be the result of lack of familial cancer visibility (FCV). Sociologists use a bias correction factor to compensate for the visibility bias which is dependent on the societal and cultural context [28, 29].

Despite the abundance of evidence regarding the lack of FCH sensitivity, widespread usage is common and it is unclear how we can improve the accuracy of FCH. Therefore, we conducted this study to investigate familial cancer visibility (FCV) as a potential determinant of FCH sensitivity in an Iranian population and to assess the hypothetical determinants of familial cancer visibility (FCV) including patients’ unawareness about her/his cancer diagnosis (PUAW) and TTND.

## Methods

### Study population

This cross- sectional study was part of an ongoing study in Iran (KCRNSU: Assessing the Completeness of Case Ascertainment of the Kerman Cancer Registry Using the Network Scale-Up Method). The main study was designed to use the network scale-up method to assess the completeness of case ascertainment of a cancer registry in a middle-sized province in Kerman, Iran.

Patients who had a confirmed malignancy (e.g., breast, colorectal, lung, leukemia, lymphoma, kidney, gastric, esophagus, ovarian, uterine, eye, sarcoma, laryngeal, liver, skin, bone marrow, thyroid, brain, bladder, testicular, bile duct, prostate or pancreatic) with the ability to speak Persian were eligible to participate in the study. A representative sample of oncology centers in Kerman was selected, including the six referral centers with the highest number of patient visits. In each center, all eligible patients were referred to our interviewers by the receptionist. Sampling was conducted during working hours in August 2014.

### Data collection & measurements

Data were collected after obtaining informed verbal consent via a face-to-face, structured interview conducted in a private room near the sampling site. Informed consent was obtained verbally because Iranians are frequently reluctant to provide written consent. If the patient consented, the verbal consent checkbox was checked by the interviewer after informing eligible patients about the study, its aims, and the study questions. All ethical considerations, including obtaining verbal consent, were approved by the Ethical Committee of Kerman University of Medical Sciences as part of approval for the KCRNSU study (KMU 9350).

All interviewers were trained in a workshop that included a role-playing interview component. Interviewees were either patients or, in cases where the patient was unaware of her/his cancer diagnosis, the most informed caregiver. If the caregiver was the interviewee, then he/she was questioned on behalf of the patient. Two questions, one for the patient’s doctor and one for the caregiver, were used to assess patients’ unawareness about her/his cancer diagnosis (PUAW). If one or both of them answered “No, the patient is not aware of his/her cancer,” that patient was coded as unaware. Interviews were gender-matched for genital cancers and for female respondents who were younger than 40. Interviews lasted around ten to fifteen minutes.

To assess FCV and TTND, an interview form was designed and validated for this study. The first section introduced the study and its aims while the second assessed PUAW, type of cancer, date of current cancer diagnosis, patients’ age, gender, marital status, and medical record code. The third section examined kinship relations using a table with relationships listed in the rows (i.e., descent or affinity (related by marriage) groups: parents, siblings and their spouses, children, grandparents, aunts and their spouses, uncles and their spouses, nephews, nieces, first cousins, spouse and his/her first-degree relatives). Table columns included the “number of living relatives,” “number of surely informed relatives," and “number of relatives who are intended to be left uninformed.” This section was modified from the standard method for visibility bias estimation [29]. Section four assessed the patients’ type of residence, monthly income, and tumor-node-metastasis (TNM) staging information. Data regarding the date of diagnosis and TNM staging of the current tumor were extracted from medical records by the patients’ oncologist.

The interview form was used in three sequential pilot studies, and several minor corrections were made regarding its validity. Its test-retest reliability was assessed around the one-month interval by 26 participants (R: mean = 0.73, 95% confidence interval (CI): 0.69, 0.76). Despite an incentive ($6.50) for the retest interview, the participation rate was 0.60.

### Data management & statistical methods

We calculated “time from diagnosis” using the date of the diagnosis and the interview date. FCV was estimated using the following formula:

$$\text{FCV}=\frac{“\text{Number of living and surely informed relatives}”}{“\text{Number of all living relatives}”}$$ (1)

TTND was defined according to the following formula:

$$\text{TTND}=\frac{“\text{Number of living relatives who were intended to be left uninformed}”}{“\text{Number of all living relatives}”}$$ (2)

The proportion of PUAW, FCV, and TTND as well as the 95% CIs were computed using the bootstrap method with 1000 resamplings. These proportions were also estimated across different subgroups based on their potential determinants.

Two or K-proportion comparison tests were used for the univariate analysis. To model potential determinants of PUAW, a mixed effects logistic regression analysis was used. Mixed effects Poisson regression was used for modeling of the FCV and TTND determinants. In both Poisson and logistic regression models, cancer type was included as a random intercept factor. To assess the significance effect of cancer type, we estimated median odds ratio (MOR) or median incidence rate ratio (MIRR) for the intercept-only models as well as for the final models.

All variables with a P-value less than 0.3 in the univariate analyses entered the multivariate model. However, those with a P-value greater than 0.05 were eliminated using a backward approach and only those with a P-value less than 0.05 were kept in the model. The stability of the models was checked using the bootstrap technique. The data analysis was conducted using Stata software (Version 11.2; StataCorp, Texas, USA).

## Results

### Participant characteristics

A total of 415 cancer patients were enrolled in the study. Participants had a mean age of 53 years (standard deviation (SD) = 15 years) and 65.5% (n = 272) were female while 34.5% (n = 143) were male. Eighty-one percent (n = 331) were from an urban area and 19% (n = 84) were from rural areas. The majority of participants (93.3%) were married (Table 1). Males were approximately 5 years older than females (P<0.001). Time from diagnosis varied from 0.1 to 280 months (mean = 28.2, SD = 40, median = 12). Time from diagnosis was significantly longer for females than males (mean = 32, SD = 44 and mean = 21, SD = 28, respectively, P = 0.009).

**Table 1:** Sampling distribution and patients’ unawareness of their cancer diagnosis.

Factor	Groups	n	Unawareness (%)	95% CI (%)	Univariate P-value
Stage at Diagnosis	I	42	12	2, 22	0.022
	II	90	17	10, 24	
	III	85	19	11, 27	
	IV	99	27	19, 36	
	Unknown^†^	60	23	16, 30	
Gender	Female	272	16	12, 21	0.64
	Male	143	27	20, 35	
Age Group	0–39	87	6	1, 10	<0.001
	40–60	187	15	9, 20	
	60+	141	36	28, 43	
Marital Status	Single	28	28	11, 43	0.299
	Married	387	20	16, 24	
Cancer Type^‡^	Breast	160	5.6	2, 9	<0.001
	Colorectal	33	44	27, 61	
	Lung	33	30	15, 46	
	Leukemia	25	28	10, 46	
	Lymphoma	28	18	4, 33	
Time from Diagnosis	Up to six M	138	27	20, 34	<0.001
	Seven to 18 M	98	20	12, 28	
	19 to 30 M	43	21	9, 33	
	31 to 42 M	34	9	0, 18	
	42+ M	102	14	7, 20	
Overall		415	20	16, 24	-

Abbreviations: CI, confidence interval; M, month.

^†^Hematologic cancers and others, the TNM data of which was not available.

^‡^First five common cancers.

### Patients’ unawareness about their cancer (PUAW)

Twenty percent (95% CI: 16%, 24%) of all patients were unaware of their cancer diagnosis. The rate of unawareness varied widely (from 5.6% (95% CI: 2%, 9%) for breast cancer to 62.5% (95% CI: 51%, 74%) for gastric cancer). Males were more likely to be unaware than females (PUAW: 27.3%, 95% CI: 20, 35; and 16.2%, 95% CI: 12, 21, respectively; P = 0.64). In the univariate analysis, the stage of tumor at diagnosis (P = 0.022), time from diagnosis (P<0.001), metastasis of tumor (P = 0.030), type of cancer (P<0.001), and age (P<0.001) were associated with PUAW. PUAW had no association with the patient’s monthly income or marital status (P = 0.32 and 0.299, respectively; Table 1). There was no significant difference in unawareness between patients from rural and urban areas (P = 0.90; Table 1).

In the adjusted multivariate model, age (crude odds ratio (OR) = 0.71, 95% CI: 0.62, 0.82; adjusted OR = 0.71, 95% CI: 0.62, 0.82) and cancer type (crude MOR = 2.16; adjusted MOR = 2.04; P<0.0001) were significantly associated with PUAW.

### The patients’ tendency for non-disclosure to relatives (TTND)

The data showed that the patients tended to withhold disclosure of their cancer from up to 16% (95% CI: 11.7, 19.4) of their relatives (Table 2). Females tended to withhold disclosure more than males (TTND = 17%, 95% CI: 12.3, 22.3 and TTND = 12%, 95% CI: 6.9, 17.5, respectively), and the lowest TTND was reported for spouses (TTND = 4%, 95% CI: 1.7, 5.4), children (TTND = 8%, 95% CI: 5.1, 11.3), parents (TTND = 13%, 95% CI: 7.9, 13.1), and siblings (TTND = 13%, 95% CI: 9.5, 17.0). Patients with lymphoma (TTND = 27%, 95% CI: 10.6, 43.7) or colorectal cancer (TTND = 21%, 95% CI: 5.0, 37.1) reported a higher TTND. Younger participants (up to 39 years) reported a higher TTND (22%, 95% CI: 11.9, 30.9) than middle-aged (14%, 95% CI: 9.4, 13.3) or elderly (12%, 95% CI: 6.5, 16.6; Table 2) participants.

**Table 2:** Familial cancer visibility and tendency for non-disclosure.

Factor	n	FCV% (95% CI)	TTND% (95% CI)
**Closeness of Relationship**			
Very close	415	88 (86, 90)	11 (7.9, 13.2)
Close	415	86 (83, 89)	15 (10.8, 18.5)
Distant	415	84 (81, 88)	19 (13.6, 24.0)
**Patient Self-Awareness**			
Yes	332	89 (86, 92)	15 (11.0, 19.5)
No	83	69 (60,78)	17 (8.5, 26.3)
**Gender**			
Female	272	87 (84, 91)	17 (12.3, 22.3)
Male	143	83 (77, 88)	12 (6.9, 17.5)
**Age Group**			
0–39	87	83 (76, 89)	22 (11.9, 30.9)
40–60	187	88 (84, 92)	14 (9.4, 13.3)
60+	141	85 (81, 89)	12 (6.5, 16.6)
**Time from Diagnosis**			
Up to six M	138	80 (74, 86)	17 (9.9, 23.1)
Seven to 18 M	98	88 (82, 93)	12 (5.6, 17.8)
19 to 30 M	43	90 (82, 98)	10 (0.3, 19.1)
31 to 42 M	34	94 (89, 99)	19 (0.3, 38.0)
42+ M	102	87 (81, 92)	19 (11.3, 26.6)
**Cancer Type** ^†^			
Breast	160	88 (83, 92)	16 (9.3, 23.4)
Colorectal	33	79 (66, 93)	21 (5.0, 37.1)
Lung	33	83 (75, 95)	15 (2.0, 27.5)
Leukemia	25	81 (67, 95)	15 (1.2, 28.5)
Lymphoma	28	82 (67, 97)	27 (10.6, 43.7)
**Overall**	415	86 (83, 89)	16 (11.7, 19.4)

Abbreviations: FCV, familial cancer visibility; TTND, tendency for non-disclosure to relatives; CI, confidence interval; M, month.

^†^First five common cancers.

Relatives’ gender, gender matching with the relatives, and stage of tumor were not included in the multivariate model (univariate P<0.3; results not shown), and the age group was ignored after adjustment (P = 0.066). Multivariate, mixed Poisson modeling showed that the patient’s gender, type of residency, marital status, monthly income, closeness to relatives, time from diagnosis, and cancer type were significantly associated with TTND (P<0.0001; Table 3). Cancer type was the most important determinant of TTND (adjusted MIRR = 14.45; P<0.0001).

**Table 3:** Determinants of cancer visibility and disclosing behavior of cancer patients.

Determinant		Crude		Adjusted		
		RR	95% CI	RR	95% CI	P-value
**Tendency for Non-Disclosure to Relatives**						
Gender (Ref: Female)		0.84	0.77, 0.91	0.87	0.76, 0.99	<0.0001
Residency (Ref: Urban)		1.74	1.58, 1.92	1.80	1.60, 2.00	<0.0001
Marital Status (Ref: Single)		0.61	0.55, 0.68	0.55	0.47, 0.64	<0.0001
Monthly Income (Ref: <100$)		0.82	0.80, 0.84	0.70	0.67, 0.73	<0.0001
Time from Diagnosis (Year)		1.02	1.01, 1.03	1.02	1.01, 1.03	<0.0001
Closeness of Relationship (Ref: Very close)	Close	1.39	1.26, 1.53	1.33	1.16, 1.52	<0.0001
	Distant	1.75	1.60, 1.92	1.41	1.23, 1.61	<0.0001
Cancer Type (Random Intercept)		15.7^†^	-	14.45^†^	-	<0.0001
**Familial Cancer Visibility**						
Patients’ Awareness (Ref: No)		1.31	1.26, 1.36	1.32	1.27, 1.38	<0.0001
Gender (Ref: Female)		0.94	0.93, 0.95	0.92	0.89, 0.95	<0.0001
TTND (25%)		0.92	0.91, 0.93	0.92	0.91, 0.93	<0.0001
Closeness of Relationship (Ref: Very close)	Close	0.97	0.94, 1.01	0.98	0.95, 1.02	0.341
	Distant	0.96	0.93, 0.99	0.96	0.93, 0.99	0.041
Age Group (Ref: 0–39 year) ^‡^		1.002	0.98, 1.02	1.03	1.01, 1.05	0.004
Cancer Type (Random Intercept)		1.20^†^	-	1.07^†^	-	<0.0001

Abbreviations: RR, rate ratio; TTND, tendency for non-disclosure to relatives; CI, Poisson confidence interval.

^†^Median incidence rate ratio for cancer type as a random intercept factor.

^‡^Age groups: 0–39, 40–60, 60+

### Familial cancer visibility (FCV)

We collected data on FCV from 27,998 relatives of cancer patients (including 3763 first-degree relatives, 8118 second-degree, 10,201 third-degree, and 5916 affinities). FCV for the total sample was estimated at 86% (95% CI: 83, 89). The rate of FCV for males and females was 83% (95% CI: 77, 88) and 87% (95% CI: 84, 91), respectively (Table 2).

Cancer was more visible in middle-aged patients (FCV = 88%, 95% CI: 84, 92) than younger (FCV = 83%, 95% CI: 76, 89) or older (FCV = 85%, 95% CI: 81, 89) patients. If patients were unaware of their diagnosis, visibility decreased from 89% (95% CI: 86, 92) to 69% (95% CI: 60, 78; Table 2).

Cancer was less visible for parents (FCV = 77%, 95% CI: 71, 83) and more visible for siblings (FCV = 90%, 95% CI: 87, 93), children (FCV = 91%, 95% CI: 88, 94), and spouses (FCV = 90%, 95% CI: 87, 93). FCV was not significantly different between second (FCV = 85%, 95% CI: 82, 89) and third-degree relatives (FCV = 85%, 95% CI: 81, 89) or affinities (FCV = 86%, 95% CI: 83, 89). Data shows that a longer time from diagnosis may lead to more FCV (P = 0.57). Breast (FCV = 88%, 95% CI: 83, 92) and lung (FCV = 83%, 95% CI: 75, 95) cancers were meaningfully more visible than colorectal (FCV = 79%, 95% CI: 66, 93) cancer (Table 2).

FCV was not significantly associated with the relatives’ gender, gender matching of patients and relatives, stage of cancer, tumor metastasis, type of residency (rural or urban), the patient’s monthly income, or type of kinship (affinity or descent) at a 0.3 significance level (results not shown). Therefore, these variables were not included in the multivariate model.

PUAW had an RR of 1.32 (95% CI, 1.27, 1.38) in the final multivariate model. Closeness of relationship (RR = 0.96, 95% CI: 0.93, 0.99), patient’s gender (RR = 0.92, 95% CI: 0.89, 0.95), and age group (RR = 1.03, 95% CI: 1.01, 1.05) as well as TTND (RR = 0.92, 95% CI: 0.91, 0.93) were significantly associated with FCV after being adjusted for other potential determinants (Table 3).

## Discussion

In this study we focused on familial cancer visibility (FCV) as a major distal determinant of family cancer history (FCH) sensitivity. Our data showed that the rates of patients’ unawareness about her/his cancer diagnosis (PUAW), Tendency for Non-Disclosure (TTND) to relatives, and FCV were 20% (95% CI: 16, 24), 16% (95% CI: 11.7, 19.4), and 86% (95% CI: 83, 89), respectively. PUAW and TTND were significantly associated with FCV in our dataset (P<0.0001). FCV was also associated with cancer type (P<0.0001), and closeness of relationship (very close vs. distant, P = 0.041), as well as patient’s age (P = 0.004), and gender (P<0.0001).

Our estimated PUAW value was meaningfully less than the values stated in some previous reports, which range from 29% to 60% [31–33], while relatively similar to values found in other reports [34, 35]. The PUAW value found in this study may be affected by our conservative PUAW measurement and the distribution of our sample. Given that PUAW is higher in end-stage and older cancer patients, and that participation in our study was not restricted to terminal or older cancer patients, the PUAW value found in our study may be lower than the values found for older, end-stage patients [32–37].

To our knowledge, this is the first study that has quantitatively assessed non- disclosure of cancer diagnosis to relatives, what we refer to as TTND. Despite using a different methodology, our results are consistent with results of available qualitative studies on TTND [26, 27]. For example, multivariate results showed that women tended to conceal their cancer diagnosis from relatives more than men (RR = 0.87; 95% CI: 0.76, 0.99), which supports qualitative results. However, this contradicts gender stereotypes (i.e., that women are more expressive than men) and may be because of the conventions of the Iranian society and more perceived stigma.

When we compared our estimated values for TTND and PUAW for different cancer types (i.e. breast cancer and gastric cancer (PUAW = 94.4%, 95% CI: 91, 98 and PUAW = 37.5%, 95% CI: 19.9, 55.2, respectively), we showed that the effect of cancer type on PUAW and TTND might be correlated with public attitudes concerning the curability of various cancers. This supports findings from other studies, such as the study by Yoshinaga et al. [38].

Again, as far as we know, this study is the first effort to estimate FCV. Given that people cannot report what they are unaware of, the estimated FCV might be interpreted as maximum sensitivity of FCHs in Kerman.

In Iran, disclosure to cancer patients is not the responsibility of physicians alone. Families and caregivers play central roles for disclosure or non-disclosure to patients and relatives [39]. In light of the central roles that families and caregivers play, the relationship between FCV and PUAW may be because a considerable proportion of families try their best to conceal cancer diagnoses from relatives in order to protect the patient from indirect disclosure. Therefore, in such families, there would be less visibility among the relatives than in families with aware patients.

Though it may be assumed that TTND and FCV are highly correlated, the obtained correlation in this study was -11% (95% Cl: 9, 13%). This weak correlation may be associated with cultural issues in the Iranian society. Information can be transmitted in two ways in Iranian families, including direct and indirect methods. During direct transmission, patients or their caregivers disclose the cancer diagnosis to relatives. Direct transmission occurs for two reasons: one, because of individual inner desires, or two, to attract financial support or other cultural incentives (regardless of inner desire). On the other hand, indirect transmission occurs when information is transmitted from those who are aware to those whom the patients or their caregivers did not intend to disclose the disease. Therefore, the association of FCV and TTND might be explained by direct information transmission.

The results of our study regarding the dependency of FCV on cancer type (MIRR = 1.07, P<0.0001) may be explained by stigmatization. However, although stigmatization may partially explain the relationship, our results are also consistent with previous studies regarding the dependency of FCH sensitivity on cancer type. For example, studies have reported high sensitivity for breast cancer, moderate sensitivity for colorectal cancer, and low sensitivity for prostate and uterine cancers [40, 41]. These findings are consistent with our estimated FCVs.

In addition, even after adjusting for confounders, FCV was lower for males than for females (RR = 0.92, 95% CI: 0.89, 0.95). This may be because men think they should conceal their weaknesses or illnesses to protect themselves and their loved ones [27]. Another possible explanation may be residual confounding caused by lower PUAW in male patients.

We also found a lower FCV in younger patients (RR = 1.03, 95% CI: 1.01, 1.05) that might decrease the accuracy and usability of FCHs. Given that numerous studies have shown that a diagnosis of cancer in younger relatives is more important for FCH than a diagnosis of cancer in older relatives, more precise investigations are needed.

In our study, FCV was highest for first-degree relatives (FDRs) (FCV = 88%, 95% CI: 86, 90). This finding was consistent with the findings for FCH sensitivity. Almost all previous studies have reported a better sensitivity for FCH in FDRs rather than second or third degree relatives [42, 43].

### Limitations

The primary limitation of our study was the result of a lower consent rate for men for participation in this study. More female participation led to a higher frequency of female-organ cancers (e.g., breast cancer). Therefore, the estimated FCV for the total sample was possibly affected by the participant sex-ratio (males = 143; females = 272). Accordingly, there is a possibility of FCV overestimation. To account for this possible overestimation, we used sampling weights extracted from Kerman Cancer Registry to weight cancer types. There was no meaningful difference (86% vs. 85.5%) between estimates from analytic or sampling weights; therefore, we presented those provided by analytic weights. In addition, given the abovementioned participant sex ratio, the overall PUAW may be underestimated. Consequently, in this study, the overall PUAW may be underestimated because of a lower PUAW in females.

Furthermore, we did not confirm interviewee claims regarding the awareness (informed or uninformed) of her/his relatives. Although four questions collecting contact information for patient relatives were originally included in the questionnaires, we omitted these questions from the finalized version since it was not feasible to collect relatives’ contact information in a pilot study. However, we used a standardized, validated method that has been used in previous sociological studies.

Finally, given the lack of a robust cancer registry or a complete archive of medical records, it was not possible for us to confirm FCHs. Accordingly, we were unable to directly estimate the effect size of PUAW, FCV, or TTND on FCH sensitivity.

## Conclusion

The maximum sensitivity of the self-reported cancer histories and FCHs in the Kermanian population is not likely to be higher than 80% and 86%, respectively. This finding might be generalizable to similar populations, given the values of PUAW and TTND. Our study suggests that improving disclosure to relatives and taking FCHs from close relatives may improve FCH sensitivity.

## Supporting Information

S1 Dataset Study dataset. (SAV)
